# FAM3B/PANDER inhibits cell death and increases prostate tumor growth by modulating the expression of Bcl-2 and Bcl-X_L_ cell survival genes

**DOI:** 10.1186/s12885-017-3950-9

**Published:** 2018-01-22

**Authors:** Paula Maciel-Silva, Izabela Caldeira, Icaro de Assis Santos, Ana Claudia Oliveira Carreira, Flavia Ramos Siqueira, Eliane Antonioli, Anna Carla Goldberg, José Ernesto Belizário, Humberto Miguel Garay-Malpartida

**Affiliations:** 1 School of Arts, Sciences and Humanities, University of São Paulo, São Paulo, SP Brazil; 2 Department of Internal Medicine, University of São Paulo School of Medicine, São Paulo, SP Brazil; 3 Department of Pharmacology, Biomedical Sciences Institute, University of São Paulo, São Paulo, SP Brazil; 4 Hospital Israelita Albert Einstein, São Paulo, SP Brazil

**Keywords:** FAM3B, Prostate cancer, Apoptosis, Cytokines, Tumor growth

## Abstract

**Background:**

FAM3B/PANDER is a novel cytokine-like protein that induces apoptosis in insulin-secreting beta-cells. Since in silico data revealed that FAM3B can be expressed in prostate tumors, we evaluated the putative role of this cytokine in prostate tumor progression.

**Methods:**

FAM3B expression was analyzed by quantitative PCR in tumor tissue clinical samples and prostate tumor cell lines. Culture growth and viability of DU145 cell line were evaluated after treatment with either exogenous FAM3B protein obtained from conditioned media (CM) of 293 T cells overexpressing FAM3B or a recombinant FAM3B protein produced in a bacterial host. DU145 cells overexpressing FAM3B protein were produced by lentiviral-mediated transduction of full-length FAM3B cDNA. Cell viability and apoptosis were analyzed in DU145/FAM3B cells after treatment with several cell death inducers, such as TNF-alpha, staurosporine, etoposide, camptothecin, and serum starvation conditions. Anchorage-independent growth in soft agarose assay was used to evaluate in vitro tumorigenicity. In vivo tumorigenicity and invasiveness were evaluated by tumor xenograft growth in nude mice.

**Results:**

We observed an increase in FAM3B expression in prostate tumor samples when compared to normal tissues. DU145 cell viability and survival increased after exogenous treatment with recombinant FAM3B protein or FAM3B-secreted protein. Overexpression of FAM3B in DU145 cells promoted inhibition of DNA fragmentation and phosphatidylserine externalization in a time and dose-dependent fashion, upon apoptosis triggered by TNF-alpha. These events were accompanied by increased gene expression of anti-apoptotic Bcl-2 and Bcl-XL, decreased expression of pro-apoptotic Bax and diminished caspase-3, −8 and −9 proteolytic activities. Furthermore, inhibition of Bcl-2 anti-apoptotic family proteins with small molecules antagonists decreases protective effects of FAM3B in DU145 cells. When compared to the respective controls, cells overexpressing FAM3B displayed a decreased anchorage- independent growth in vitro and increased tumor growth in xenografted nude mice. The immunohistochemistry analysis of tumor xenografts revealed a similar anti-apoptotic phenotype displayed by FAM3B-overexpressing tumor cells.

**Conclusions:**

Taken together, by activating pro-survival mechanisms FAM3B overexpression contributes to increased resistance to cell death and tumor growth in nude mice, highlighting a putative role for this cytokine in prostate cancer progression.

**Electronic supplementary material:**

The online version of this article (10.1186/s12885-017-3950-9) contains supplementary material, which is available to authorized users.

## Background

Prostate cancer is a heterogeneous disease that can exhibit various degrees of aggressiveness and metastasis patterns, and variable response to therapy. Therefore, there is a great need to identify molecular prognostic factors that allow stratification of prostate cancer patients in groups that are more homogeneous [1]. Furthermore, the identification of new molecular targets leading to new therapeutic approaches is clearly needed for the effective diagnosis, treatment, and prognosis of metastatic prostate cancer.

Pancreatic-derived factor (FAM3B/PANDER, or simply FAM3B), is a novel secreted protein discovered through in silico screening for novel cytokine families based on a highly conserved four-helix bundle secondary structure [2, 3]. FAM3B was primarily localized to the endocrine pancreas and identified in both α- and β-cells [4, 5]. Initial characterization evaluating the role of FAM3B on pancreatic islets revealed induction of pancreatic β-cell apoptosis via caspase- 3 and cyclin-dependent kinase inhibitor 1A (p21) pathways, suggesting that FAM3B is a potential activator in a setting of type 1 diabetes [6]. However, recent evidence using a FAM3B knockout mouse model revealed another biological role for this protein in the regulation of glycaemia via regulation of liver and pancreas functions [7]. Additional studies have demonstrated that FAM3B is significantly regulated by both glucose and insulin, supporting a biological role in glycemic control and regulation of lipogenesis [8–12].

Several public databases have confirmed the hypothesis that FAM3B is a pleiotropic cytokine that exerts multiple cellular functions in pancreas and liver and, likely, pathological effects when overexpressed in breast and prostate tumor tissues. Interestingly, FAM3C (or ILEI), another FAM3 family member, is highly expressed in patients with prostate cancer [13]. In order to comprehend further the role of this cytokine in prostate tumor progression, we evaluated the effects of FAM3B overexpression in the androgen-insensitive DU145 prostate tumor cell line. We demonstrated that, in contrast to its pro-apoptotic role in pancreatic β-cells, FAM3B was capable of inhibiting TNF-α-mediated programmed cell death pathways by increasing the expression of anti-apoptotic Bcl-2 family members and diminishing the caspase-3 proteolytic activity. Together these results suggest FAM3B affects prostate tumorigenesis by modulating the expression of the cell survival genes Bcl-2 and Bcl-X_L_.

## Methods

### Reagents

Recombinant human TNF-α was purchased from Peprotech, (Mexico DF, Mexico). DMEM medium, fetal bovine serum, MTT reagent, staurosporine, camptothecin, and propidium iodide were obtained from Sigma (St. Louis, MO, USA). FITC-Annexin-solution was kindly provided by Dr. Gustavo Amarante-Mendes (Department of Immunology, ICB-USP, Brazil). Ac-DEVD-AMC antibody, caspase-3 specific substrate and the pan-caspase inhibitor zVAD-FMK were purchased from Calbiochem (San Diego, CA, USA). Power Sybr®Green master mix was purchased from Applied Biosystems (San Diego, CA, USA). All PCR primers were purchased from BioNEER (Great Seneca Highway Rockville, MD, USA). Recombinant human FAM3B (rhFAM3B) produced in bacterial hosts was obtained from Novus Biologicals (Littleton, CO, USA). Rabbit polyclonal antibodies against Bcl-2, Bax, Bcl-X_L_, Caspase-3, Caspase-8, and Caspase-9 for western blot analysis were purchased from Cell Signaling (San Diego, CA, USA). Mouse monoclonal anti-β-actin and anti-Bax antibodies and rabbit polyclonal anti-Bcl-2 antibodies for immunohistochemical analyses were purchased from Santa Cruz Biotechnology (Santa Cruz, CA, USA). Mouse monoclonal anti- PCNA (Proliferating Cell Nuclear Antigen) was obtained from Dako (Glostrup, Denmark). Mouse monoclonal antibody against human FAM3B was obtained from R&D Systems (Minneapolis, MN, USA). Anti-rabbit and anti-mouse IgG HRP-linked antibodies were purchased from Cell Signaling (San Diego, CA, USA). All other chemicals of analytical grade were obtained from E. Merck & Co., Inc. (Darmstadt, Germany). Obatoclax Mesylate (GX15–070), an antagonist of Bcl-2 and ABT-737, a BH3-mimetic pan-inhibitor of Bcl-xL, Bcl-2, and Bcl-w were purchased from Sigma (St. Louis, MO, USA).

### Cell culture and treatments

LnCAP, PC-3, and DU145 prostate tumor cells, and the 293 T embryonic kidney immortalized cells were originally obtained from the American Type Culture Collection (ATCC, Rockville, MD) and were cultured in DMEM medium supplemented with 10% fetal bovine serum (FBS, Hyclone, Logan, UT, USA), 10 U mL^−1^ penicillin, and 10 U mL^−1^ streptomycin (Life Technologies, Gaithersburg, MD), at 37 °C in a humidified atmosphere containing 5% CO_2_. For functional assays, DU145 cells were seeded at a density of 5 × 10^5^ cells/mL (except in the MTT assays) and subsequently cultured in presence/absence of several cell death inducers as described below, in order to measure cell viability and cell death.

### Cloning of FAM3B cDNA into lentiviral and plasmidial vectors

Human FAM3B was amplified by RT-PCR from human pancreatic islets mRNA. The PCR primers specifically targeting the cDNA encoding the full-length sequence of FAM3B, including *Bam*HI and *Eco*RI restriction sites (underlined) were as follows: 5’-GGATCCCAAGATCTCCAAGGATTCG-3′ (forward) and 5’-GAATTCTTTTTACAGATGCTTTCAG-3′ (reverse). PCR products were purified from agarose gels after electrophoretic separation and cloned into pGEM-T vector (Promega, Madison, WI). Clones having appropriately sized inserts after digestion with restriction enzymes were sequenced using the T7 promoter primer (ABI PRISM Big Dye Terminator cycle sequencing kit, Applied Biosystems, Branchburg, NJ). Subsequently, this cDNA was cloned into *Bam*HI and *Eco*RI sites of the pCSC-SP-PW lentiviral mammalian expression vector (Addgene plasmid 12,335, kindly provided by Dr. Ricardo Garcia Correa) to produce pCSC-FAM3B plasmid for lentivirus production. Subsequently FAM3B cDNA was cloned into pcDNA 3.1 mammalian expression vector (Life Technologies, CA, USA) by using the same restriction sites. pcDNA-FAM3B plasmid was used to produce FAM3B secreted protein in conditioned media of 293 T cell cultures.

### Recombinant human FAM3B protein assays

In order to verify the effects of exogenous FAM3B on cell viability 1 × 10^6^ DU145 cells were seeded onto 2 cm diameter dishes and treated with recombinant human FAM3B protein (rhFAM3B) obtained in bacterial host. Because TNF-α alone did not induce death in DU145 cells [14, 15], TNF-α-induced activity was enhanced by the addition of cycloheximide (CHX), a protein synthesis inhibitor. Cells were maintained in two experimental conditions: group 1 was cultured under non-stimulated proliferation conditions (DMEM media +0.5% SFB) and group 2 was treated with several TNF-α doses (0–20 ng/mL) + CHX (1 mM). After 12 h cells were treated with rhFAM3B: group 1 cells with several concentrations (0–1000 pM) and group 2 cells only with 1000 pM. Cell viability was monitored by MTT assay after 5 days and 24 h, respectively. All experiments were performed in triplicate and were repeated at least three times independently.

### Conditioned media assays

293 T cells were plated at a density of 5 × 10^6^ cells/ in 10-cm-diameter dish and transfected with pcDNA3-FAM3B or pcDNA-empty vectors, in OptiMEM I media using Lipofectamine 2000)® (Life Technologies, CA, USA). Media was changed 4 h after transfection to equal volumes of DMEM media +10% fetal bovine serum (SFB). After 24 h, conditioned media (CM) was harvested, cleared to remove cell debris, and either used immediately or stored at −80 °C. The FAM3B secretion in the CM was verified by western blot. After 12 h in culture, 1 × 10^6^ DU145 cells were treated with CM in several dilutions (1:1, 1:10, 1:100) and cell viability/death was monitored at 24, 48, and 96 h by MTT assay and DNA fragmentation. CM from transfected cells with pcDNA-empty vector was used as control.

### Lentivirus-mediated transduction in DU145 prostate tumor cells

Lentiviral particles were produced by using a four-plasmid transfection system (pCSC-FAM3B or PCSC-empty plasmids with three other packaging plasmids) in the 293 T cell line, as described previously [16, 17]. After transient transfection, the lentivirus-containing supernatants were collected, filtered, and concentrated by centrifugation. DU145 cells were infected with lentivirus particles in the presence of 4 mg/mL polybrene (Sigma, St. Louis, MO, USA). After infection, expression of FAM3B was confirmed at mRNA and protein levels by qRT-PCR and western blot, respectively. DU145 cells infected with virus carrying empty vector were used as control in all subsequent experiments.

### Cell viability

DU145 cells were seeded on 96-well microtiter plates (1 × 10^4^/well) and treated with following death inducers: a) 2 ng/mL TNF-α + 1 μM cycloheximide (CHX), b) 1 μΜ staurosporine (STS), c) 20 nM camptothecin (CMP), d) 50 μM Etoposide (ETO), and e) serum starvation medium (0.05% SFB). Cell treatment at various times was followed by addition of 0.05 μg/mL of *3 (4,5-dimethylthiazol- 2-yl)-2,5-diphenyltetrazolium bromide)* (MTT) and 2 h incubation at 37 °C. After a brief centrifugation, supernatant was carefully removed and 100 μL DMSO was added to each well. After insoluble crystals were completely dissolved, absorbance at 570 nm was read on a ThermoMax microplate reader (Molecular Devices, Sunnyvale, CA).

### DNA fragmentation

DU145 cells were seeded on 6-well microtiter plates (3 × 10^5^/well) and treated with 0.2, 2.0, and 20 ng/mL TNF-α + 1 μM CHX. After 48 h of treatment cell culture medium was collected and centrifuged to harvest floating dead cells. The adherent cells were harvested by trypsinization and mixed with the previously collected cells. After centrifugation at 1000 g × 5 min, cells were rinsed twice with phosphate-buffered saline (PBS) and lysed in a hypotonic buffer, pH 7.4, containing 50 μL/mL propidium iodide (PI) (Invitrogen), sodium citrate 0.1% m/v, and Triton X-100 0.1% m/v. Cellular DNA fragmentation was measured on a FACS-Calibur ® flow cytometer using the CellQuest ® program (BD, San Jose, CA, USA).

### Apoptosis/necrosis index

After harvesting, approximately 1 × 10^6^ DU145 cells were washed once with ice-cold PBS followed by annexin-V binding buffer (10 mM HEPES, 140 mM NaCl, and 2.5 mM CaCl_2_, pH 7.4). Cells were then resuspended in binding buffer and incubated with the fluorescein isothiocyanate-labeled annexin-V solution (FITC-annexin) (1:500 *V*/V in binding buffer) and 10 μg/mL PI for 15 min at room temperature in the dark. Cells were washed again with annexin-V binding buffer and immediately analyzed by flow cytometry.

### RNA extraction and cDNA synthesis

Total RNA extracted from clinical samples of prostate adenocarcinoma tissues (*N* = 19) and their control non-neoplasic tissue (*N* = 28) were kindly provided by Dra. Dirce Carraro from the A.C. Camargo Hospital Biobank. All samples have signed informed consent for use in research, provided and approved by patients and approved by the institutional research ethics committees. Total RNA from dissected tumors of xenotransplanted mice (1–2 mg) and cell cultures (3 × 10^5^ cells/mL) was extracted with TRIzol™ method (Invitrogen, Carlsbad, CA, USA) according to the manufacturer’s instructions. Quality assessment of mRNA was made by agarose gel analysis of 18S and 28S rRNA bands integrity. 1–2 μg of RNA per sample were treated with RQ1 RNase-Free DNase (Promega, Madison, WI, USA) and cDNA was synthesized by using the SuperScript™ III Reverse Transcriptase kit (Invitrogen, CA, USA), with random and *OligodT* primer mixture, according to the manufacturer’s instructions.

### Real-time quantitative RT-PCR (qRT-PCR)

Real-time PCR was performed according to the Power Sybr®Green protocol (Applied Biosystems), using the Sequence Detector ABI PRISM 5700, (Perkin-Elmer/Applied Biosystems, Foster City, CA). The nucleotide sequences specific for qRT-PCR gene amplification were as follows: FAM3B 5’-CCAAAATCCCTGCTCTTCATG-3′ (forward) and 5’GCATTCTTGGCATCGTTATTCA-3′ (reverse); Bcl-2 5’-CTGGGATGCCTTTGTGGAA-3′ (forward) and 5’-CAGCCAGGAGAAATCAAACAGA-3′ (reverse); Bax 5’CAAGAAGCTGAGCGAGTGTC-3′ (forward) and 5’-GAAGTTGCCGTCTGCAAACA-3′ (reverse); Bcl-X_L_ 5′- CAGACCCAGTGAGTGAGCAG-3′ (forward) and 5’CCGGTTGCTCTGAGACATTT-3′ (reverese); HPRT 5’-GAAGGTCTTGCTCGAGATGTG-3′ (forward) and 5’-TCCAGCAGGTCAGCAAAGAAT-3′ (reverse). The primers were designed to span an intron within the cDNA sequence target, making the cDNA amplification product easily distinguishable from the genomic product. We used a 2-step amplification protocol with a denaturation temperature of 95 °C and an annealing-extension temperature of 60 °C. Relative gene expression was calculated from cycle threshold values (*Ct*) using the formula 2^–ΔΔCt^ [18]. The human HPRT gene was used as internal control for each individual sample gene expression.

### Caspase activity

Approximately 5 × 10^5^ DU145 cells were resuspended in hypotonic lysis buffer (10 mM HEPES, 50 mM NaCl, 2 mM EDTA, 5 mM DTT, 0.1% CHAPS, 1 mM PMSF, and 10% Sucrose, pH 7.4) and stored at –80 °C. The lysate was then subjected to four freeze-thaw cycles before centrifugation at 10,000 g for 10 min. Protein concentrations were determined in the supernatants by the Bradford colorimetric assay (Biorad, USA). The reaction was started at 37 °C by incubating 100 μg of total protein with caspase-specific substrates (caspase-3, DEVD-AFC; caspase-8, IETD-AFC; caspase-9: LEHD-AFC) in assay buffer (0.05 M HEPES, pH 7.4, 10 mM DTT, 100 mM NaCl, 1 mM EDTA, 0.1% CHAPS, 10% sucrose) and allowed to incubate at 37 °C for 45 min. Protease activity was monitored every 30 s within a total period of 30 min at an excitation wavelength of 400 nm and an emission wavelength of 510 nm using a SpectraMax Gemini XPS spectrofluorometer (Molecular Devices, CA, USA). Caspase activity was calculated per microgram of protein, and expressed as a percentage of control activity. Assays were performed in the presence or the absence of the pan-caspase inhibitor 1 μM ZVAD-FMK as control for broad specificity of caspase activity.

### Bcl-2 protein family inhibition assays

We performed inhibition assays for Bcl-2 and Bcl-XL in order to verify the contribution of these proteins to the anti-apoptotic effect of FAM3B in DU145 cells. We used two specific inhibitors to perform the experiments: a) Obatoclax Mesylate (GX15–070), an antagonist of Bcl-2, that also acts upon Mcl-1; b) ABT-737, a BH3-mimetic pan-inhibitor of Bcl-xL, Bcl-2, and Bcl-w, with no inhibition observed against Mcl-1. We used these inhibitors alongside TNF-α (1 and 5 ng /ml) + 1 μM CHX to confirm the role of Bcl-2 and Bcl-X in FAM3B-mediated cell death resistance to TNF-α.

### Western blot

Approximately 1 × 10^6^ cells or 50 mg of tumor tissue were harvested and sonicated in 100 μL of ice-cold lysis buffer (20 mM HEPES, pH 7.4, 1 mM EDTA, 150 mM NaCl, 1% NP-40, 10 mg/mL aprotinin, and 1 mM PMSF). An aliquot was taken for total protein determination according to the Bradford method. Proteins were precipitated by the addition of 3 volumes of cold acetone and pelleted by centrifugation at 12000 g for 10 min. Pellets were solubilized in SDS-β-mercaptoethanol sample buffer by boiling for 4 min. Equal amounts of protein (50–100 μg) were run on 7.5–12% SDS-polyacrylamide gels and electrically transferred to nitrocellulose membranes. After wash, membranes were then incubated overnight with the primary antibodies in PBS with 15% fat-free milk-powder, at working dilutions for the following rabbit polyclonal antibodies against: caspase-3 (1:1000), caspase-9 (1:2000), caspase-8 (1:1000), Bcl-2 (1:200), Bax (1:500) and Bcl-X_L_ (1:500) and mouse monoclonal antibodies against FAM3B (1:500), and β-actin (1:1000). After wash with washing buffer (1X TBS, 0.1% Tween-20) membranes were incubated with secondary antibodies horseradish peroxidase-linked goat anti-rabbit IgG (1:5000) or horseradish peroxidase-linked horse anti-mouse IgG (1:2500) for 2 h at room temperature under agitation. The Enhanced Chemiluminescence System (ECL; Amersham-Pharmacia, Buckinghamshire, UK) was used for detection according to the manufacturer’s instructions. Immunoblot results were quantified by using *ImageJ* image processing software (freely available at https://imagej.nih.gov/ij/index.html) and normalized by β-actin expression.

### Soft agar assays

Cells were plated in 6-well plates using a two-layer soft agar system with 1 × 10^3^ cells per well in a volume of 1 mL per well as described earlier [19]. In brief, a 2.5% agarose stock was prepared in PBS. The bottom 0.6% agar support was prepared in DMEM containing 10% FBS. Cells were harvested, washed, and mixed with the top-agarose suspension at a final concentration of 0.3%, which was then layered onto the bottom agar. The agar plates were incubated at 37 °C changing the medium every 3 days. After 21 days, cells were fixed with 3.7% formaldehyde and then the size and number of colonies was determined using an inverted microscope. All experiments were repeated independently at least three times using triplicate plates.

### Xenografts in nude mice

Animal care and experiments were approved by the Animal Care and Use Committee of the Biomedical Institute of São Paulo University, according to the Brazilian Society of Experimental Biology guidelines. Male 6- to 8-week-old nude (nu/nu) mice were obtained from Biomedical Sciences Institute of University of Sao Paulo (Brazil). Exponentially growing 1 × 10^6^ DU145/FAM3B and DU145/control cells were mixed with 200 μL of Matrigel® and injected subcutaneously into the dorsal region of nude mice. After the first week, primary tumor growth was monitored twice a week by measuring the tumor diameter with calipers and calculating tumor volume (mm^3^) using the standard formula V = (L + W^2^)/2, where L is the length and W is the width of the tumor mass. Tumors were harvested at varying times thereafter for molecular and immunohistochemical assays.

### Histology and immunohistochemistry analyses

Nude mice were sacrificed after 6–8 weeks. Dissected tumors were fixed in formalin (10%) and embedded in paraffin for subsequent histology analysis with haematoxilin-eosin (HE). Immunohistochemistry was performed on 4-μm-thick sections mounted on glass slides precoated with 2% silane. Sections were deparaffinized and rehydrated by conventional techniques, then heated in citrate buffer for antigen retrieval, pretreated with 30% hydrogen peroxide in methanol for endogenous peroxide blockade and preincubated with normal horse serum diluted at 1:70 in nonfat milk at 2% in Tris-buffered saline (TBS) to prevent nonspecific binding. Sections were then incubated overnight at 4 °C with anti-Bax, anti-Bcl-2 antibodies, and anti-PCNA, diluted at 1:100, in a solution containing 1% BSA diluted in TBS. The EnVision Labelled Polymer for peroxydase (Dako Glostrup, Denmark) was used before development with 3-Amino-9-Ethyl-Carbazol (AEC) or DAB substrate. All sections were counterstained with Harri’s hematoxylin and covered with DAKO Glycergel® (Dako Glostrup, Denmark). For negative control experiments, incubation with the primary antibody was not performed.

### Statistical analysis

Data are presented as the mean ± standard deviation (SD). Each experiment was repeated at least three times with triplicate values within each group. Differences between means were analyzed by one-way ANOVA and Student’s t-test. A *p* value <0.05 was considered statistically significant. Analyses were performed using the *GraphPad Prism* software version 5.1 (San Diego, CA, USA).

## Results

### FAM3B is expressed by human prostate tumors and prostate tumor cell lines

**Fig. 1:**
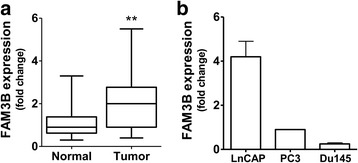
FAM3B expression in human prostate tumors and prostate tumor cell lines. *FAM3B* gene expression was analyzed at mRNA level by qRT-PCR in (**a**) Tumor clinical samples (*N* = 19) and non-neoplastic tissue clinical samples (*N* = 29) and (**b**) LnCAP, PC-3 and DU145 prostate tumor cells. The results are expressed as means of three independent experiments. In tumor clinical samples statistical differences were determined by Student’s t-test (*p < 0.05, **p < 0.01)

We evaluated FAM3B expression at mRNA level in prostatic normal tissue and tumor samples as well as in prostate tumor cell lines. Our results showed that FAM3B expression was upregulated in 13/19 (70%) prostate adenocarcinoma samples compared to 11/29 (38%) non-neoplastic tissue (Fig. 1a). Relative mRNA expression levels in the normal prostate tissues and primary prostate tumors ranged from 0.30 to 3.30 (mean 1.07 ± 0.12) fold and from 0.40 to 2.77 (median, 2.28 ± 0.30) fold, respectively (*p* = 0.00095). FAM3B was expressed at high levels in androgen-dependent LnCAP cells but at low levels in the androgen-insensitive PC-3 and DU145 cells (Fig. 1b). We then chose DU145 cells to evaluate the effects of treatment with exogenous FAM3B protein or FAM3B produced by lentiviral-mediated overexpression system.

### Exogenous treatment with FAM3B increases cell viability of DU145 cells

**Fig. 2:**
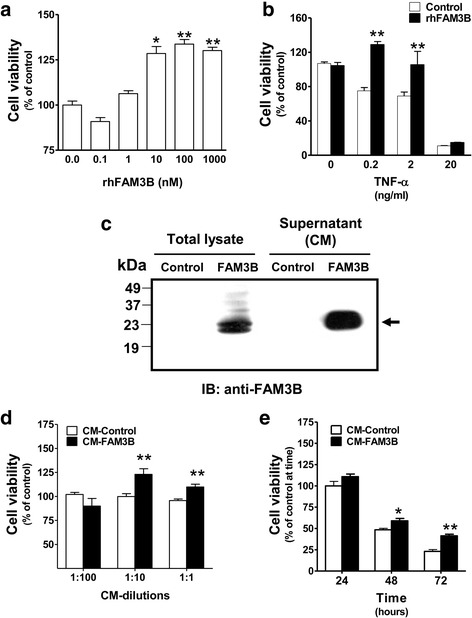
Exogenous rhFAM3B and CM-FAM3B enhance cell viability of DU145 cells. Analysis of cell viability was performed in DU145 cells treated during 48 h with several concentrations of (**a**) rhFAM3B (0.1–1000 pM); (**b**) rhFAM3B (100 pM) + TNF-α (added at a final concentration as indicated) + CHX (1 mM). **c** Western blot of total cell lysate and supernatant (culture medium +0.5% SFB) from 293 T cells transfected with pcDNA-FAM3B plasmid. The anti-FAM3B antibody recognized cytoplasmic and secreted FAM3B protein in culture medium (CM-FAM3B). Cell viability of DU145 cells was measured after (**d**) 48 h with several dilutions of CM-FAM3B and (**e**) 24, 48 and 72 h with a 1:10 dilution of CM-FAM3B. The results are expressed as means of three independent experiments and as relative ratios to the CM and recombinant protein-free controls (* *p* < 0.05; ** *p* < 0.01)

Since FAM3B is a cytokine-like protein, we decided to evaluate paracrine effects of this recombinant protein on growth of DU145 cells. As shown in Fig. 2a, DU145 cells displayed a relative dose-dependent increase in cell viability following 48 h exposure to recombinant human FAM3B (rhFAM3B) produced in bacterial host. This increase in cell viability was particularly evident following 10 pM rhFAM3B treatment. However, non-morphological changes were noted after rhFAM3B addition to cultures. When TNF-α-dependent cell death is induced in DU145 cells, rhFAM3B (1 nM) had a protective effect even at a 2 ng/mL dose of TNF-α (Fig. 2b). In order to verify the effects of FAM3B obtained from a eukaryotic host, we produced FAM3B protein in human 293 T cells transiently transfected with the pCSC-FAM3B plasmid. We achieved high yields of FAM3B protein in both whole lysate and conditioned media (Fig. 2c). In whole lysate, the specific anti-FAM3B antibody recognized two different sized bands of ~ 23 kDa and ~ 25 kDa. However, we found that the secreted protein in the media consisted of a single band of ~ 23 kDa.

As measured by MTT assay, an increase of nearly 30% and 20% in DU145 cell viability was achieved with 1:10 and 1:1 dilutions of conditioned media containing secreted protein (CM-FAM3B), respectively, when compared with conditioned media from empty vector transfected cultures (CM-control). No advantage was observed at the 1:100 dilution (Fig. 2d). When time-dependence was evaluated, increase in cell viability reached 20% and 40% after 48 h and 72 h in culture with CM-FAM3B, respectively (Fig. 2e).

### FAM3B overexpression protects DU145 cells from TNF-α-induced apoptosis

**Fig. 3:**
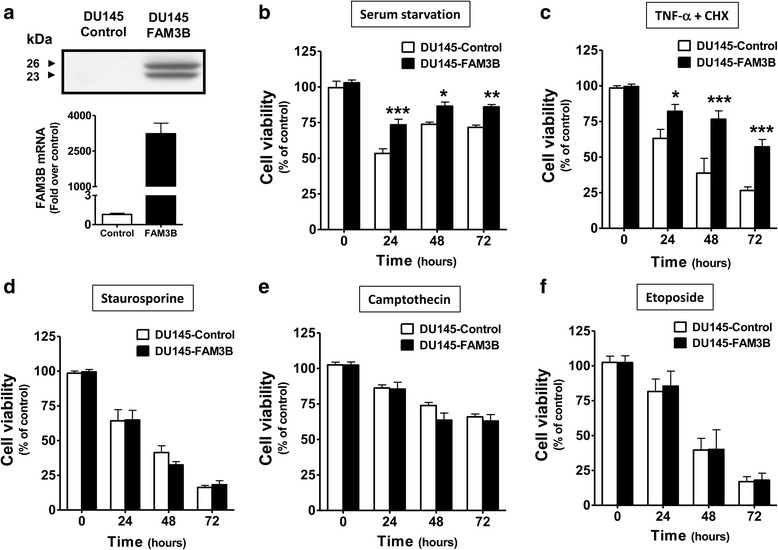
FAM3B overexpression increases cell viability of DU145 cells upon TNF-α-induced apoptosis. **a** FAM3B expression was measured by RT-PCR (lower graphic) and Western blot (upper graphic) in DU145 cells after infection with a lentivirus carrying the pCSC-FAM3B plasmid. Cell viability was measured by MTT assay in DU145/FAM3B cells treated during 24, 48, and 72 h with (**b**) serum starvation medium (0.05% SFB), (**c**) 2 ng/mL TNF-α + 1 μM cycloheximide (CHX), (**d**) 1 μΜ staurosporine (STS), (**e**) 20 nM camptothecin (CMP), (**f**) 50 μM Etoposide (ETO). The results are expressed as means of three independent experiments and as relative ratios to cell viability measured in DU145-control cells (* *p* < 0.05; ** *p* < 0.01)

Lentivirus-mediated FAM3B overexpression in DU145 cells was confirmed by qRT-PCR and western blot. By RT-PCR we calculated a ~2000 fold increase of FAM3B mRNA after infection with pCSC-FAM3B-derived lentiviruses (upper graph, Fig. 3a). Western blot analysis again showed two different-sized molecules of 23 kDa and 25 kDa (lower graph, Fig. 3a). Overexpression of FAM3B in DU145 cells slightly increased growth rate but had no effect on cell proliferation, as showed by growth curve and BrdU incorporation (Additional file 1: Figure S1). In accordance with the effects previously observed with recombinant and secreted FAM3B, DU145/FAM3B cells were resistant to cell death induced by serum starvation conditions (Fig. 3b) and TNF-α + CHX (Fig. 3c). We did not observe the same response when cell death was induced by the kinase inhibitor staurosporine (Fig. 3d), DNA-damage drug camptothecin (Fig. 3e), or etoposide (Fig. 3f), all of which act on DNA synthesis and cell cycle arrest, but not through TNF-α mediated apoptotic cell death.

**Fig. 4:**
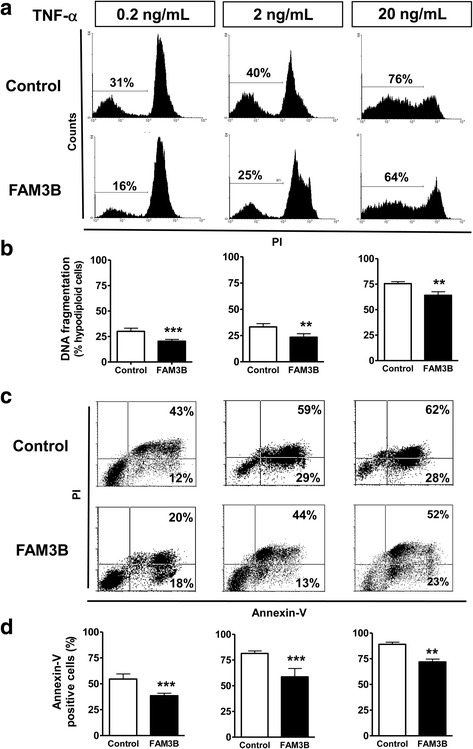
Overexpression of FAM3B decreases DNA fragmentation and membrane exposure of phospholipid phosphatidylserine (PS). **a** Flow cytometric analysis of DNA fragmentation by propidium iodide (PI) staining of DU145 cells upon treatment with TNF-α (0.2 ng/mL, 2 ng/mL and 20 ng/mL) + CHX for 24 h. Hypodiploid cells are considered as DNA-fragmented cells (apoptotic cells) shown on the left side of the representative histograms. **b** Cells overexpressing FAM3B show lower rates of DNA fragmentation than control cells. Y-axis of bars shows mean rate ± SE of fragmented cells in three independent experiments. **c** Identification of PS exposure by annexin-V-FITC staining of DU145 cells following treatment with TNF-α (0.2 ng/mL, 2 ng/mL and 20 ng/mL) + CHX for 24 h. Representative dot plots show annexin-V positive cells (lower right quadrant) and annexin-V + PI positive cells (upper right quadrant), summing total apoptotic cells (**d**). Cells overexpressing FAM3B have lower rates of PS exposure than control cells. Y-axis of bars shows mean rate ± SD of total annexin-V positive cells in three independent experiments. Statistical differences were determined by Student- t test. (* *p* < 0.05; ** *p* < 0.01)

Flow cytometric analysis of DNA fragmentation using propidium iodide staining showed that FAM3B was able to inhibit apoptosis in DU145 cells after a 24 h treatment with several doses of TNF-α + CHX (Fig. 4a-b). The maximum anti-apoptotic effect (nearly 30%) is reached at lower TNF-α doses (0.2 ng/mL). To confirm these effects we examined whether FAM3B secretion inhibited phosphatidylserine (PS) externalization by annexin V-FITC binding assay. In agreement with DNA fragmentation data, FAM3B inhibited most efficiently the PS externalization at lower doses of TNF-α, reaching similar 30% inhibition of cell death at the dose of 0.2 ng/mL (Fig. 4c-d). Therefore, the protection measured in both assays was similar to those obtained with rhFAM3B and CM-FAM3B proteins. Taken together, the results confirm the protective effects of FAM3B after TNF-α-induced cell death.

### Bcl-2 and Bcl-X_L_ anti-apoptotic proteins are up regulated by FAM3B in DU145 cells

**Fig. 5:**
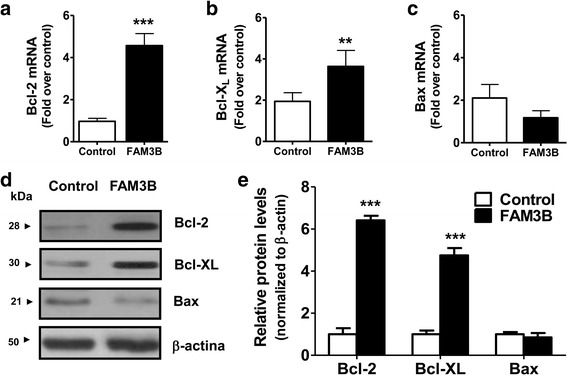
Expression of Bcl-2 and Bcl-X_L_ anti-apoptotic genes correlates with FAM3B overexpression. Gene expression analysis at mRNA level in DU145/FAM3B cells by quantitative RT-PCR for (**a**) Bcl-2, (**b**) Bcl-X_L_, and (**c**) Bax. Y-axis of bars shows mean ± SD fold increase in gene expression compared to control cells, in three independent experiments. HPRT housekeeping gene was included as a normalizer control. **d** Representative images from western blotting analysis with specific antibodies for *FAM3B,* Bax, Bcl-2, Bcl-X_L_, and *β-actin,* which was included as a loading control. Lanes were obtained from the same membrane and were contiguous, except in second row (Bcl-X_L_). **e** The relative intensity of bands was calculated by densitometry and was assumed as gene expression at protein level. Y-axis of bars shows mean ± SD fold increase of relative protein level, in three independent experiments. In all cases, statistical differences were determined by Student’s t-test (*p < 0.05; **p < 0.01)

To investigate the molecular mechanisms underlying apoptosis inhibition by FAM3B we conducted various analyses of gene expression to measure pro- and anti-apoptotic genes. At mRNA level FAM3B overexpression induced a ~5 fold increase in Bcl-2 expression (Fig. 5a), a ~4 fold increase in Bcl-X_L_ expression (Fig. 5b) and a slight decrease in the pro-apoptotic Bax expression (Fig. 5c). In accordance with these results, western blot analyses confirmed an increased Bcl-2 (~7 fold) and Bcl-X_L_ (~5 fold) expression in DU145/FAM3B cells, but no change in Bax protein levels (Fig. 5d-e). These data suggest that the intrinsic apoptotic pathway is involved in the effects of FAM3B on DU145 prostate tumor cells.

### Inhibition of Bcl-2 anti-apoptotic family proteins decreases protective effects of FAM3B

**Fig. 6:**
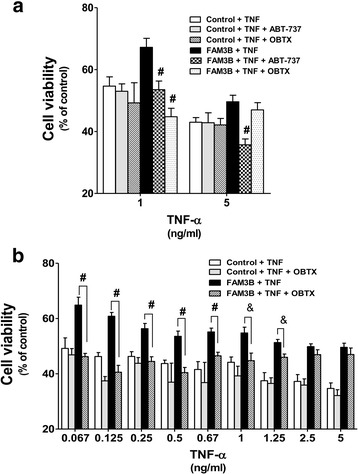
Inhibition of Bcl-2 anti-apoptotic family proteins decreases protective effects of FAM3B. **a** DU145 cells were treated with 1 ng/ml and 5 ng/ml TNF-α + 10 μM CHX and 10 μM ABT-737or 0.1 μM GX15–070 (OBTX) (**b**) a dose-dependent assay between 0.13 and 5 ng/ml TNF and 0.1 μM GX15–070 (OBTX). The results are expressed as means of three independent experiments and as relative percent of control cells or FAM3B cells respectively. Statistical differences were determined by Two-way Anova test. (# *p* < 0.001; & *p* < 0.01)

Following treatment with both TNF-α doses and 10 μM ABT-737, a statistically significant decrease in the resistance against TNF-α + CHX-induced cell death was observed in DU145-FAM3B cells. On the other hand, 0.1 μM GX15–070 (OBTX) only decreased resistance to cell death when 1 ng/ml TNF-α was used (Fig. 6a). These data confirm the key role of Bcl-2 and Bcl-XL in FAM3B-mediated protection against cell death in DU145 cells. In a dose-dependent assay we determined that in the presence of 2 ng/ml TNF-α, GX15–070 (OBTX) does not have any significant effect (Fig. 6b).

### FAM3B overexpression reduces caspase-3, −8 and −9 proteolytic activities upon TNF-α stimulation

**Fig. 7:**
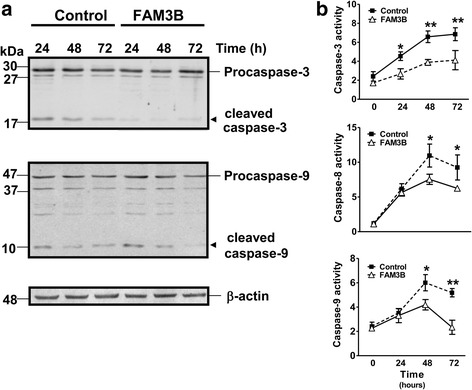
Caspase activity is reduced in DU145/FAM3B cells. **a** Representative images from western blotting analysis with specific antibodies for caspase-3 (upper image) and caspase-9 (middle image) normalized by β-actin expression (bottom image) in cells treated with 2 ng/mL TNF-α + CHX at time indicated. Arrows indicate the active form of caspase-3 and -9 which is related to level of caspase proteolytic activity. **b** Caspase proteolytic activity measured by enzymatic assays with synthetic substrates for caspase −3 (upper bar graph), caspase-9 (middle graph bar) and caspase-8 (bottom graph bar). Y-axis shows relative fluorescence units (RFU) at each time point as indicated. Data shown are mean ± SD of three independent experiments. The differences were measured by two-way ANOVA test (*p < 0.05; **p < 0.001; *** < *p* 0.0001)

To confirm anti-apoptotic effects of FAM3B in DU145 cell, we measured the activation of caspase-8 and caspase-9, both initiator caspases, and caspase-3, the major effector caspase, by using specific flourogenic substrates and a western blot assay. Initially, after FAM3B overexpression, no changes were observed (data not shown). However, when apoptosis was induced with TNF-α (2 ng/mL), we observed a decreased activation of caspase-3 and -9 (Fig. 7a) as measured by western blot analysis. The cleaved form of caspase-3 was diminished up to 24 h after TNF-α-treatment (Fig. 7a, upper panel) and the active form of caspase-9 (10 kDa) decreased 48 h after TNF-α treatment in DU145/FAM3B cells (Fig. 7a, low panel). Furthermore, fluorogenic cleavage assays confirmed that caspase-3, −8 and −9 activities were lower in DU145- FAM3B cells after TNF-α treatment, when compared to control cells (Fig. 7b), suggesting that the inhibitory effects of FAM3B on cell death are mediated, at least in part, by caspase-dependent mechanisms.

### FAM3B promotes in vitro tumorigenicity and increases tumor growth in nude mice

**Fig. 8:**
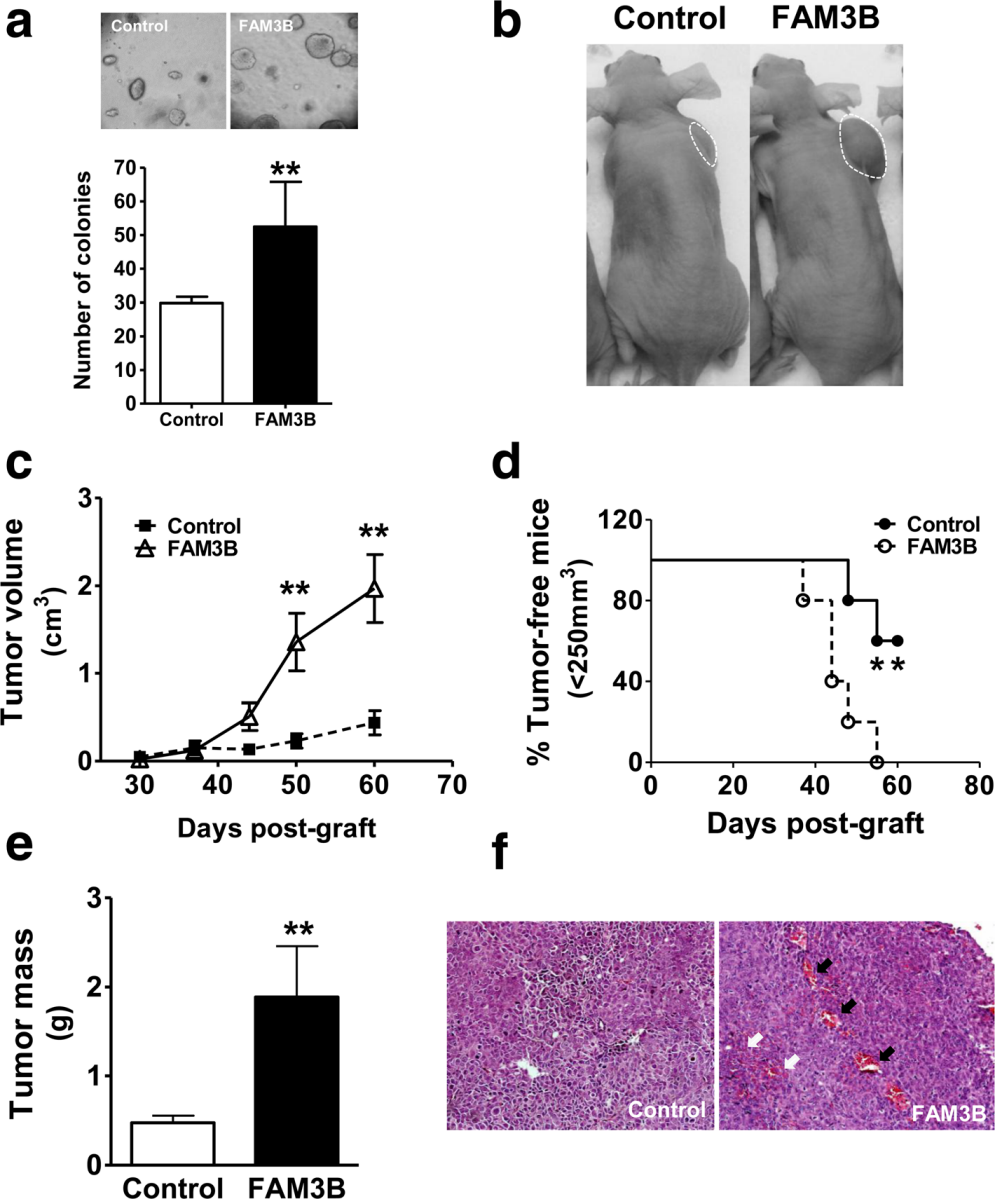
FAM3B overexpression increases tumorigenicity in vitro and tumor growth in nude mice. **a** Soft agar assay of colony formation, after cell culture during 21 days. FAM3B overexpressing DU145 cells led to the formation of larger and more abundant colonies when compared to control cells (upper panel). After Coomassie blue staining, number of colonies formed per well was quantified and plotted in the Y-axis bar (**b**) Representative mouse showing the largest tumor size observed in DU145/FAM3B animals as compared with a representative control animal. **c** Tumor growth was accompanied during 60 days, with tumor volume measured in time intervals as indicated. **d** Kaplan-Meier survival analysis exhibits the slightly accelerated tumor induced by FAM3B expression. **e** After dissection, tumors from mice were weighed and compared (**f**). HE analysis shows DU145/FAM3B tumors have more blood vessels (black arrows) and more hemorrhagic sites (white arrows) than control tumors suggesting a more angiogenic phenotype. All quantitative data are expressed as mean ± SD of tumor volumes of 3–5 animals in each experimental group, in three independent experiments. Statistical differences among groups were determined by Student’s t-test and one-way ANOVA test with Tukey’s pairwise comparisons (*p < 0.05, **p < 0.01)

To evaluate the effects of FAM3B overexpression in tumorigenicity of DU145 cells, we performed the soft agar assay for colony formation, which evaluates the growth in anchorage-independent conditions. FAM3B overexpressing DU145 cells led to the formation of larger colonies and increased ~2 fold the number of soft agar colonies (Fig. 8a) when compared to DU145/control cells. These data suggested that FAM3B might affect the seeding of tumors. We next asked whether FAM3B could enhance the in vivo tumorigenicity. DU145/FAM3B and DU145/control cells were grafted subcutaneously onto the dorsal region of nude mice (3–5 animals per experimental group). Tumor development was measured at regular intervals using calipers to assess tumor volume. When compared to control cells, DU145/FAM3B cells rapidly developed bigger and heavier tumors within six weeks (Fig. 8b-c) with a slightly accelerated tumor onset (Fig. 8d). In addition, the final tumor weight measured after 8 weeks demonstrated an overall significant (~4 fold, *p* < 0.05) increase in the size of tumors derived from DU145/FAM3B cells (Fig. 8e). Surprisingly, microscopic examination by HE analysis showed that DU145/ FAM3B tumors had a larger amount of blood vessels with bleeding more visible than the control tumors (Fig. 8f), suggesting an angiogenic phenotype, which is essential to tumor progression and metastasis. Taken together, results indicate FAM3B overexpression may further enhance the tumorigenicity and even promote a malignant phenotype of established prostate tumor cells. It remains to be seen if this observation is due to secreted FAM3B protein by DU145/FAM3B cells.

### Increased tumor growth was correlated with the expression of FAM3B and anti-apoptotic proteins

**Fig. 9:**
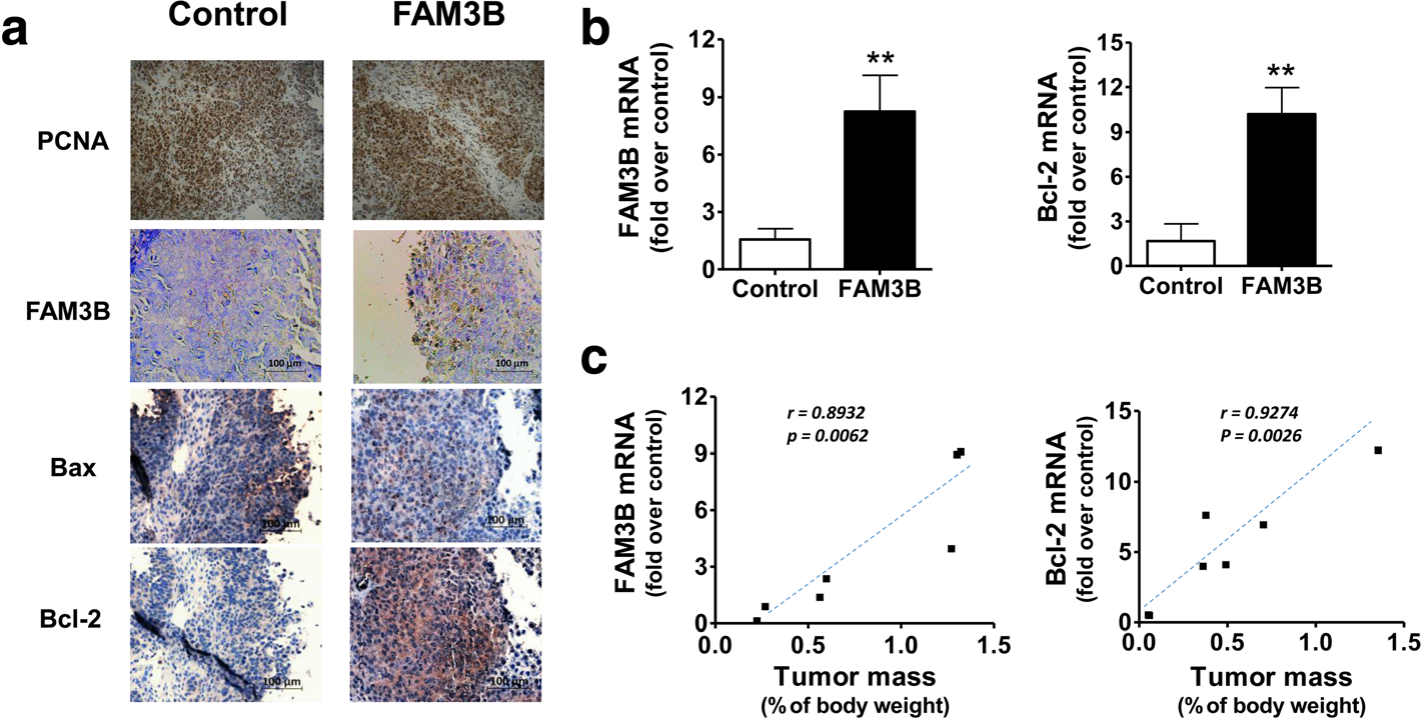
Increased tumor growth correlates with the expression of FAM3B and anti-apoptotic proteins. **a** Representative images of immunohistochemistry showing the expression of PCNA, FAM3B, Bax and Bcl-2 proteins in tumor xenografts tumors derived from DU145/FAM3B and DU145/control cells, stained in dark-brown. **b** Quantitative RT-PCR genes showing FAM3B overexpression in tumor samples (right) accompanied by increased mRNA Bcl-2 gene expression (left). Y-axis bars show mean ± SD fold increased gene expression compared to the mean of control tumors, normalized by expression of the HPRT housekeeping gene, in three independent experiments. Statistical differences among groups were determined by Student’s t-test (*p < 0.05, **p < 0.01). **c** Pearson’s correlation test shows a high and statistically significant correlation between tumor mass (% body weight) and FAM3B and Bcl-2 gene expression (fold increase in gene expression). For this comparison we included the tumor mass of all animals. When *r* > 0.8 and *p* < 0.05, we considered correlation to be highly positive

In order to identify mechanisms underlying the tumor growth promotion by FAM3B in nude mice, we performed gene expression analysis by RT-PCR and immunohistochemistry in dissected tumor samples. As demonstrated by PCNA staining we verified that FAM3B overexpression do not induces cell proliferation, in agreement with in vitro data, but promotes an antiapoptotic phenotype by upregulating Bcl-2 anti-apoptotic and decreasing Bax pro-apototic gene expression, at protein (Fig. 9a) and mRNA (Fig. 9b) levels. Moreover, we observed a statistically significant correlation when comparing the tumor mass from animals with FAM3B gene expression (*r* = 0.8932; *p* = 0.0062) and Bcl-2 gene expression (*r* = 0.9274; *p* = 0.0026) in all animals (Fig. 9c). This data are suggesting that tumor growth is driven by a FAM3B-mediated diminished cell death rather than increased cell proliferation. Thus, these data highlight the role of Bcl-2 family proteins in cell survival activated by FAM3B in prostate tumor growth.

## Discussion

FAM3B was initially detected in the endocrine pancreas and named PANDER (PAncreatic DERived factor) because it was secreted and shown to control the synthesis and metabolic actions of insulin and glucose and was likely to have a role in lipogenesis [2, 9, 12]. In this study we show, for the first time, the role of FAM3B in tumor cell death, tumor growth, and invasiveness in prostate cancer.

First, we verified the increased expression of FAM3B in clinical samples of prostate adenocarcinoma and three of the most representative tumor cell lines used in prostate cancer research (LnCAP, PC3 and DU145). Furthermore, we show that both recombinant FAM3B (rhFAM3B) and conditioned medium containing the secreted protein (CM-FAM3B) increase cell viability alone or in combination with TNF-α + CHX in DU145 prostate cell line. Paradoxically, FAM3B was recognized as an inducer of apoptosis in pancreatic β-cells. In those studies recombinant FAM3B and overexpression of FAM3B diminished cell viability and induced cell death in a βTC3 insulinoma cell line, in a dose and time-dependent manner [3, 4]. Others showed that FAM3B acts as a regulator of the cyclin-dependent kinase inhibitor CDKN1A and activates caspase-3 [6]. On the other hand, we speculated initially that the apparently opposite effects operated by FAM3B in DU145 cells were due to cell proliferation rather than to an anti-apoptotic mechanism because similar results had been previously described for IL-6 in prostate cancer tumors [13, 20]. Nonetheless, the absence of a proliferative phenotype after FAM3B overexpression (Additional file 1) confirmed that, in contrast to its pro-apoptotic role in pancreatic β-cells, FAM3B has an anti-apoptotic role in prostate tumor cells. However, the possibility that apoptosis may not be the sole cell death mechanism inhibited by FAM3B in these cells has still to be investigated.

In agreement with results from the FAM3B exogenous treatment, FAM3B overexpressing DU145 cells (DU145/FAM3B) showed increased cell viability after serum starvation (0.5% SFB) and/or treatment with 2 μg/mL of TNF-α, when compared to DU145/control cells, confirming the protective effect of FAM3B. However, after treatment with STS, a potent inhibitor of a variety of protein kinases that induces apoptosis in DU145 cells in dose-dependent manner [21, 22], we did not observe the same effect even after a 72 h treatment with 1 μM STS. A possible explanation as to why FAM3B failed to inhibit STS-mediated cell death is that Bcl-2 is not involved STS-induced apoptosis [22]. We therefore chose to employ TNF-α, an agonist of the extrinsic pathway to investigate the anti-apoptotic role of FAM3B in these cells.

Nuclear fragmentation and phosphatidylserine externalization were inhibited in FAM3B/DU145 cells treated with different doses of TNF-α. Moreover, since FAM3B/ DU145 cells were capable of secreting this protein into the culture medium we expected an autocrine effect would inhibit TNF-α-induced cell death. Our results suggest that overexpressing FAM3B promotes survival in DU145/FAM3B cells through increased Bcl-2 and Bcl-X_L_ expression and inhibition of caspase-3 cleavage. It is known that TNF-α can trigger both NF-κB and apoptosis signaling simultaneously, so suppression of TNF-α-induced NF-κB signaling by CHX is expected to potentiate TNF-α induced apoptosis through caspase-3, whereas overexpression of Bcl-2 and Bcl-X_L_ would inhibit this same activation [14, 23]. Although the inhibition of caspase activity was observed only after TNF-α treatment it is reasonable to propose that FAM3B might directly or indirectly upregulate the anti-apoptotic caspase inhibitors, such as survivin, with ensuing resistance to cell death. Indeed, it has been already demonstrated that inhibition of the key molecules Bcl-2, Bcl-XL, and survivin is needed to sensitize DU145 cells to TNF-α-induced apoptosis [24, 25]. Since GX15–070 can also inhibit the anti-apoptotic protein Mcl-1 [26], we concluded that there are different mechanisms of cell death being mediated by Bcl-2 and Mcl-1, at low doses of TNF-α + CHX in DU145 cells. On the other hand, it is well known that different doses of TNF induce different effects on prostate tumors [27], reinforcing the hypothesis that FAM3B may inhibit different mechanisms and types of cell death in DU145 cells, including autophagy and necrosis as suggested [28]. This hypothesis should be further addressed in future studies.

On the other hand, the inhibition of upstream caspase-8 does not appear to be a key step for the FAM3B anti-apoptotic effects in the DU145 cells as this inhibition was observed only 48 h after TNF treatment.

The inhibition of cell death supports the hypothesis that PANDER exerts a role in the development and progression of tumors by increasing invasiveness and tumorigenicity. Our findings showed that overexpression of FAM3B promotes in vitro tumorigenicity in DU145 cells and increases tumor growth in mice suggesting a role for FAM3B in prostate tumor progression. In addition, immunohistochemical data indicates that, at least in part, FAM3B promotes tumorigenicity in these cells by the upregulation of Bcl-2 and downregulation of Bax, with a high correlation between tumor growth and FAM3B/Bcl-2 gene expression. Interestingly, similar findings were reported in colon carcinoma HCT8 cells [29]. In summary, our results show that overexpression of FAM3B leads to increased expression of Bcl-2 and Bcl-XL, which results in the inactivation of caspases and decreases the rate of DU145 cells undergoing apoptosis, consequently promoting an increased tumor growth.

The presence of several blood vessels and hemorrhagic features in tumors as well as micrometastases detected exclusively in lungs from animals injected with DU145/FAM3B cells suggests that FAM3B may also promote angiogenesis, which plays a major role in a metastatic tumor phenotype. Interestingly, the transcription factor SLUG is targeted by FAM3B to promote epithelium mesenchymal transition (EMT) in colon carcinoma cells and inhibition of FAM3B by RNAi is associated to decreased Bcl-2 in colon tumor cells [29, 30]. SLUG has been described as a survival factor in neuroblastoma, lung, and esophageal carcinomas by upregulating Bcl-2 [31–33], therefore, SLUG can be the transcription factor that linked FAM3B and Bcl-2 increased expression in DU145 cells. Furthermore, we attempted to establish an association of FAM3B-induced upregulation of Bcl-2 and Bcl-XL with the invasive phenotype of DU145/FAM3B cells. Interestingly, the association of increased levels of Bcl-2 and Bcl-XL expression and the progression from localized to disseminated stages using an androgen-independent prostate cancer phenotype has been established previously [34–37]. Recent studies recognized Bcl-2 as a biomarker for prognosis of prostate cancer [38] and others showed association of high Bcl-2 expression with higher Gleason scores and lower survival in patients with advanced prostate cancer [39]. Thus, we conclude that FAM3B-mediated upregulation of Bcl-2 anti-apoptotic family members can contribute to apoptosis inhibition and, consequently, increased of tumor growth in mice xenotransplanted with DU145 cells. Therefore, FAM3B may become a promising molecular target for diagnosis and therapy of prostate cancers.

## Conclusion

In the present study we confirm the anti-apoptotic effect of FAM3B in prostate tumor cells, which is in agreement with functions of FAM3B in colorectal tumors. In summary, our findings reveals, for the first time, that increased expression and secretion of FAM3B in vivo may play an important role in development and progression of prostate tumors mainly by increasing Bcl-2 and Bcl-X_L_ cell survival genes.

## Additional file

Additional file 1: Figure S1. Cell proliferation assays in DU145/FAM3B cells (**A**) Viable DU145/FAM3B and DU145-control cells were harvested and counted under a light microscope using the trypan blue exclusion method at 48 h intervals during 15 days to determine growth curves. The growth curves shown represent data from three separate experiments. (**B**) Cell proliferation was measured by labeling cells with bromodeoxyuridine (BrdU) incorporation assay kit according to manufacturer’s protocol. The results are expressed as means of three independent experiments and as relative ratios to BrdU incorporation in DU145-control cells. (TIFF 2263 kb)

## Abbreviations

ANOVA: Analysis of variance
CHX: Cycloheximide
CM: Conditioned media
CMP: Camptothecin
DMEM: Dulbecco’s modified eagle’s medium
DTT: Ditiotreitol
EDTA: Ethylenediamine tetraacetic acid
EMT: Epithelium mesenchymal transition
ETO: Etoposide
FITC: Fluorescein isothiocyanate
HE: Hematoxylin-eosin
HEPES: 4-(2-hydroxyethyl)-1-piperazineethanesulfonic acid
HRP: Horseradish peroxidase
IgG: Immunoglobulin G
mRNA: Messenger RNA
MTT: 3-(4,5-dimethylthiazol-2-yl)-2–5-diphenyl tetrazolium bromide
PANDER: PANcreatic-DERived factor
PBS: Phosphate-buffered saline
PCR: Polymerase chain reaction
PI: Propidium iodide
PS: Phosphatidylserine
qPCR: Quantitative polymerase chain reaction
RFU: Relative fluorescence units
RNA: Ribonucleic acid
RT: Reverse transcription
SBF: Fetal bovine serum
SD: Standard deviation
STS: Staurosporine
TBS: Tris-buffered saline
TNF-α: Tumor necrosis factor
zVAD-FMK: Carbobenzoxy-valyl-alanyl-aspartyl-[O-methyl]- fluoromethylketone
