# Supplementary figures and images for: FAM3B/PANDER inhibits cell death and increases prostate tumor growth by modulating the expression of Bcl-2 and Bcl-XL cell survival genes
Source: BMC Cancer. 2018 Jan 22;18:90. doi: 10.1186/s12885-017-3950-9 (PMC5778767; doi:10.1186/s12885-017-3950-9)

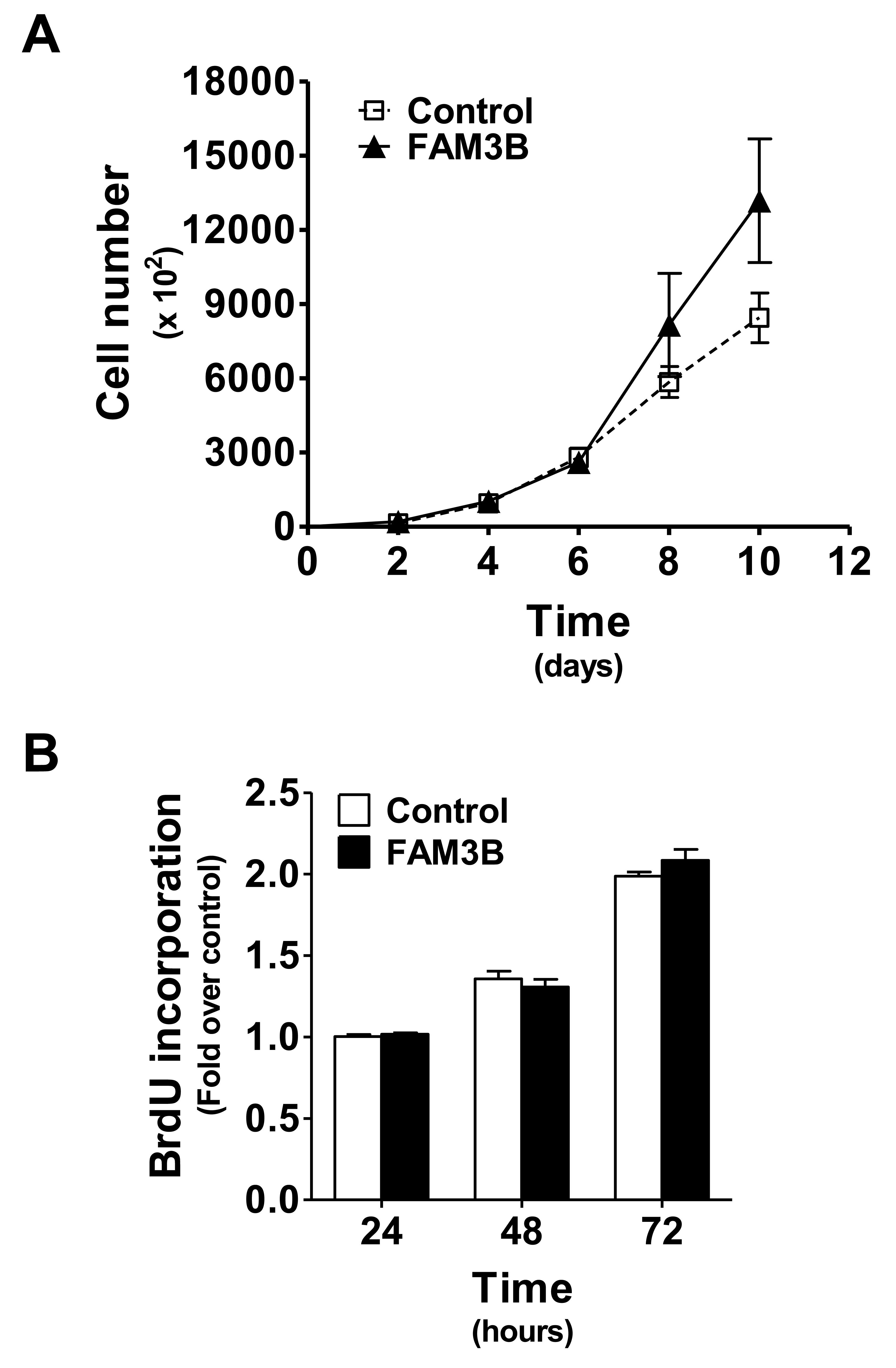

Supplement: Additional file 1: Figure S1. — Cell proliferation assays in DU145/FAM3B cells (A) Viable DU145/FAM3B and DU145-control cells were harvested and counted under a light microscope using the trypan blue exclusion method at 48 h intervals during 15 days to determine growth curves. The growth curves shown represent data from three separate experiments. (B) Cell proliferation was measured by labeling cells with bromodeoxyuridine (BrdU) incorporation assay kit according to manufacturer’s protocol. The results are expressed as means of three independent experiments and as relative ratios to BrdU incorporation in DU145-control cells. (TIFF 2263 kb) [file 12885_2017_3950_MOESM1_ESM.tif]
